# Sequencing Biological and Physical Events Affects Specific Frequency Bands within the Human Premotor Cortex: An Intracerebral EEG Study

**DOI:** 10.1371/journal.pone.0086384

**Published:** 2014-01-17

**Authors:** Fausto Caruana, Ivana Sartori, Giorgio Lo Russo, Pietro Avanzini

**Affiliations:** 1 Brain Center for Social and Motor Cognition, Italian Institute of Technology, Parma, Italy; 2 Department of Neuroscience, University of Parma, Parma, Italy; 3 Claudio Munari Center for Epilepsy Surgery, Ospedale Niguarda-Ca’ Granda, Milan, Italy; University Medical Center Groningen UMCG, Netherlands

## Abstract

Evidence that the human premotor cortex (PMC) is activated by cognitive functions involving the motor domain is classically explained as the reactivation of a motor program decoupled from its executive functions, and exploited for different purposes by means of a motor simulation. In contrast, the evidence that PMC contributes to the sequencing of non-biological events cannot be explained by the simulationist theory. Here we investigated how motor simulation and event sequencing coexist within the PMC and how these mechanisms interact when both functions are executed. We asked patients with depth electrodes implanted in the PMC to passively observe a randomized arrangement of images depicting biological actions and physical events and, in a second block, to sequence them in the correct order. This task allowed us to disambiguate between the simple observation of actions, their sequencing (recruiting different motor simulation processes), as well as the sequencing of non-biological events (recruiting a sequencer mechanism non dependant on motor simulation). We analysed the response of the gamma, alpha and beta frequency bands to evaluate the contribution of each brain rhythm to the observation and sequencing of both biological and non-biological stimuli. We found that motor simulation (biological>physical) and event sequencing (sequencing>observation) differently affect the three investigated frequency bands: motor simulation was reflected on the gamma and, partially, in the beta, but not in the alpha band. In contrast, event sequencing was also reflected on the alpha band.

## Introduction

Classic studies of the monkey brain have shown that the premotor cortex (PMC) contains a repertoire of motor primitives representing meaningful motor acts such as grasping, tearing or reaching, or temporal fragments of these acts such as the opening/closing phases in grasping [1] [2]. According to imaging studies in humans, a role of the PMC is to organize the ordinal structure of these motor acts to produce complex motor sequences [3] [4] [5]. In addition to motor coding, the PMC also plays a role in a number of non-executive cognitive functions, including action understanding [6], motor imagery [7] [8], conceptual knowledge for actions [9] and the processing of action-related language [10]. All these cognitive functions are classically explained in terms of the reactivation of the motor primitives stored in the PMC, decoupled from their executive functions, and exploited for different purposes by embodied motor simulations [11] [12].

The evidence that PMC also contributes to cognitive tasks in which the action representation is not directly involved, such as the sequencing of observed events, challenges this framework. Imaging studies have shown that the PMC is activated during the sequencing of different types of structured events and, most importantly, that the activation is independent from the biological or non-biological nature of the stimuli [13] [14] [15]. In fact, while the premotor activation observed during action sequencing can easily be explained in terms of motor simulation, in contrast the same explanation can hardly stand for its activation during the sequencing of non-biological events. However, some attempt to link event sequencing to action representation has been done. The most relevant theoretical framework has been given by Schubotz [16] in her Habitual Pragmatic Event Map (HAPEM) model. According to this model the difference between the sequencing and prediction of reproducible events and that of irreproducible, non-biological, events is smaller than it seems because the structure of the non-biological event is transformed and mapped into the motor system, by exploiting an audiomotor or visuomotor representation. In other words, the motor system is exploited in a simulation mode to predicts some of the relevant dynamics of the observed event. Despite we find the embodied account for event sequencing proposed by HAPEM very promising, and agree that event and action sequencing could both exploit the sensorimotor system, however some important differences between the two processes must be stressed. In particular, as also noticed by Schubotz [16], action sequencing differs considerably to event sequencing for the fact that only actions instantiate goals and intentions, thus suggesting that the sequencing of actions requires, beside those processes involved in event sequencing, also the detection of a goal that the action is aimed at. Furthermore, while action sequencing is possibly based on a motor imagery, thus involving the covert stages of action planning such as kinematics, in contrast event sequencing certainly does not depend on a motor imagery strategy. As a consequence, given the vicinity of the motor imagery strategy to motor preparation [7] [17], the activation of the PMC during the sequencing of biological action must be somehow different to that during the sequencing of physical events. The aim of the present study was to investigate how motor simulation and event sequencing coexists within the same cortical region, and how these mechanisms interact when both functions are executed. More specifically, to provide new insights that are unavailable to imaging techniques, our interest was to investigate whether the sequencing of biological and non-biological events are differently coded, in different frequency bands. To this purpose we intracranially recorded from drug-resistant epileptic patients with depth electrodes implanted in the PMC the modulation of the alpha (8–13 hz), beta (15–30 hz) and gamma (50–150 hz) bands during a sequencing task involving both biological and non-biological events. More specifically, we asked subjects to passively observe a randomized arrangement of images depicting biological actions (OBS-BIO) and physical events (OBS-PHY) and, in a second block, to sequence them in the correct order (SEQ-BIO and SEQ-PHY, respectively). This paradigm allowed us to investigate the possible existence of an event sequencing mechanism in PMC, eliciting a stronger modulation during the sequencing task as compared to the passive observation of the same stimuli (SEQ > OBS). Furthermore, it also allowed to investigate the possible existence of a motor simulation mechanism, eliciting a stronger modulation during the processing of the biological stimuli as compared to the physical events (BIO > PHY). In particular, according with the expectancy that a motor simulation strategy is required to solve the actions sequencing [18] [19], we expected the motor simulation to be more evident in the sequencing task only, that is, SEQ-BIO > SEQ-PHY.

An interesting insight comes from the direct comparison of the modulation in the alpha and beta frequency bands, commonly available also to scalp EEG studies, to the reactivity of the high-gamma frequency band. The broadband frequency interval 50–150 Hz here investigated is a typical feature of intracranial recordings in epileptic patients, while scalp EEG has no access to such a high frequency range. The broadband gamma frequency is of particular interest since it is currently associated to neuronal population spiking activity [20] [21], correlate with the BOLD signal [22] [23], and is spatially and functionally more specific than the power modulation in other bands. Furthermore, activity in the gamma band show poor overlap with responses in the lower frequency bands as well as with intracranial ERP [24] [25] [26]. Recent intracranial EEG studies from patients provided evidence of a gamma modulation in a series of cognitive functions such as spatial attention, reading, gaze coding [25] [26] [27] [28], but poor is known on its modulation during the covert stages of action planning and motor simulation. As a consequence, since the gamma frequency band reliably correlates with local neuronal activity, and the localization of the alpha and beta frequency band generators within the motor system is still debated, we compared their task-dependent activity in the light of the mu-rhythm literature.

## Methods

### Participants

The experiment was performed on eight patients (F = 2; M = 6, age 24 ±5; Right = 6; Left = 2) suffering from drug-resistant focal epilepsy and stereotactically implanted with intracerebral electrodes as part of their pre-surgical evaluation, at the “Claudio Munari” Center for Epilepsy Surgery, Ospedale Niguarda-Ca’ Granda, Milan, Italy. Implantation sites were selected on purely clinical grounds, on the basis of seizure semiology, scalp-EEG and neuroimaging studies, and with no reference to the present experimental protocol. Patients were fully informed of the electrode implantation and stereo-EEG recordings, and, according to the Declaration of Helsinki (BMJ 1991; 302: 1194) gave written informed consent to participate in the study. Experimental procedures were approved by the Ethical-Scientific Committee of the Ospedale Niguarda-Ca’ Granda. We selected patients whose precentral region was not affected by epileptic activity. No seizures were recorded during the 24 hours prior to the experiment. No alteration in the sleep/wake cycle was observed, and no additional pharmacological treatment was applied before the experiment. Patients did not show any motor or cognitive deficits during the examination; they fully understood the instructions and easily performed the experimental task.

### Electrode Implantation

For each patient, up to fifteen depth electrodes were implanted in different regions of the brain including the precentral region. To reach the clinically relevant targets, the stereotactic coordinates of each electrode were calculated preoperatively based on the individual’s cerebral MRI. Each electrode had a diameter of 0.8 mm and was comprised of 10–15 2 mm long contacts, spaced 1.5 mm apart (DIXI®, Besancon, France). Cerebral structures explored by each electrode contact were determined by coregistration of pre-implantation volumetric brain MRI with post-implantation volumetric brain CT, and visualized by a software package for visualization and image analysis (3DSlicer®; see [25]).

### Procedure

**Figure 1:**
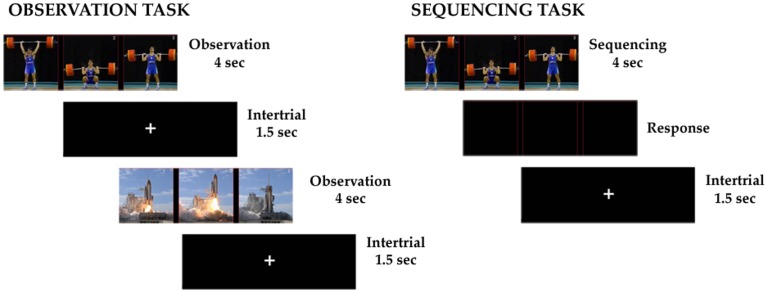
Experimental paradigms for the “observation task” (left) and “sequencing task” (right). In both tasks, stimulus presentation lasted 4sec. Only in the “sequencing task” the stimulus was followed by the command to digit on the keyboard the correct sequence. for Both sequencing and observation tasks had an intertrial period of 1.5sec, followed by a new trial. In the left panel two consecutive trials are shown, one representing a biological action and the other representing a physical event.

Recordings were obtained in a dimly-light quiet room. The patient was seated approximately 100 cm from the laptop display where stimuli were presented. The stimuli consisted of three pictures extracted from a single movie and presented simultaneously in a horizontal array (Figure 1). The spatial distribution of the pictures was random, i.e. they were not necessarily in the correct order. Pictures were extracted from 60 movies, depicting biological actions (n = 30) and physical events (n = 30). Biological movies represented human actions familiar from everyday life. Since the target region was independent of the experimental task, it was impossible to know in advance the functional properties of the final location of the contacts. Therefore, to be as inclusive as possible, half of the biological movies showed whole body movements (such as sport scenes), while the others showed movements of the hands and forearms (such as manual abilities). Physical movies represented dynamic scenes involving physical objects, such as a plane take-off or a space shuttle launch. The same set of stimuli was presented in two blocks. During the first block (Observation block, OBS) the patient was asked to passively observe the images, without any specific task to accomplish. Images were presented for 4sec and then followed by an intertrial period of 1.5sec. At the end of the OBS, the patient was informed that the pictures presented in each trial were extracted from single movies and randomly distributed in the horizontal axes. In the second block, the same set of stimuli was presented and the patient was asked to detect the correct sequence of the three pictures (Sequencing block, SEQ). Images were presented for 4sec, as in OBS, and were followed by the command to digit on the keyboard the correct sequence, using the hand ipsilateral to the implanted hemisphere (Figure 1). No temporal constraint was given for the answer of the patient. An intertrial period of 1.5sec started at the end of the patient response. Behavioral results were recorded for the analysis. Sixty trials per condition (biological and physical) were recorded in both OBS and SEQ. The instruction to passively observe was always given in the first block, while the instruction to make a judgment only in the second block. This block design was imposed to rule out the possibility that the patient performed the sequencing task also in OBS, that is, when the sequencing was not requested. In fact, given our main interest in the SEQ-BIO vs. SEQ-PHY comparison, the passive observation task was mainly aimed to rule out that possible differences between the biological and physical events sequencing were due to low-level features of the stimuli.

### Stereo-EEG Recording and Analysis

During the experiment continuous stereo EEG (SEEG) was recorded with a 1000 Hz sampling rate by means of a 192 channel-EEG device (EEG-1200 Neurofax, Nihon Kohden®). Each channel was referred to a contact in the white matter far from the recording sites, in which low and high frequency electrical stimulations did not produce any subjective or objective manifestation (neutral reference). At the end of each experimental session, SEEG data were exported and the activity of each contact located in the precentral region (n = 107 recording sites) was selected. A visual inspection was carried out by clinicians in order to ensure the absence of any pathological interictal activity. Trials showing artifacts were removed. A band-pass filter (0.015–500 Hz) was applied to avoid any aliasing effect. Each trial was epoched with a [–1500, +4500] ms time window, with respect to the image onset. Activity in the alpha (8–13 Hz), beta (15–30 Hz) and gamma (50–150 Hz) frequency bands were analyzed in the time-frequency (TF) domain by convolution with complex Morlet’s wavelet. According to previous intracranial studies [24] [25] gamma power was estimated for 10 adjacent non overlapping frequency bands, each 10 Hz wide, and a divisive baseline correction was applied versus the prestimulus interval for each single band [–500/0]. Conversely, alpha and beta power were computed with a single frequency band exploring, respectively, the frequency ranges 8–13 Hz and 15–30 Hz.

### Statistical analysis

**Figure 2:**
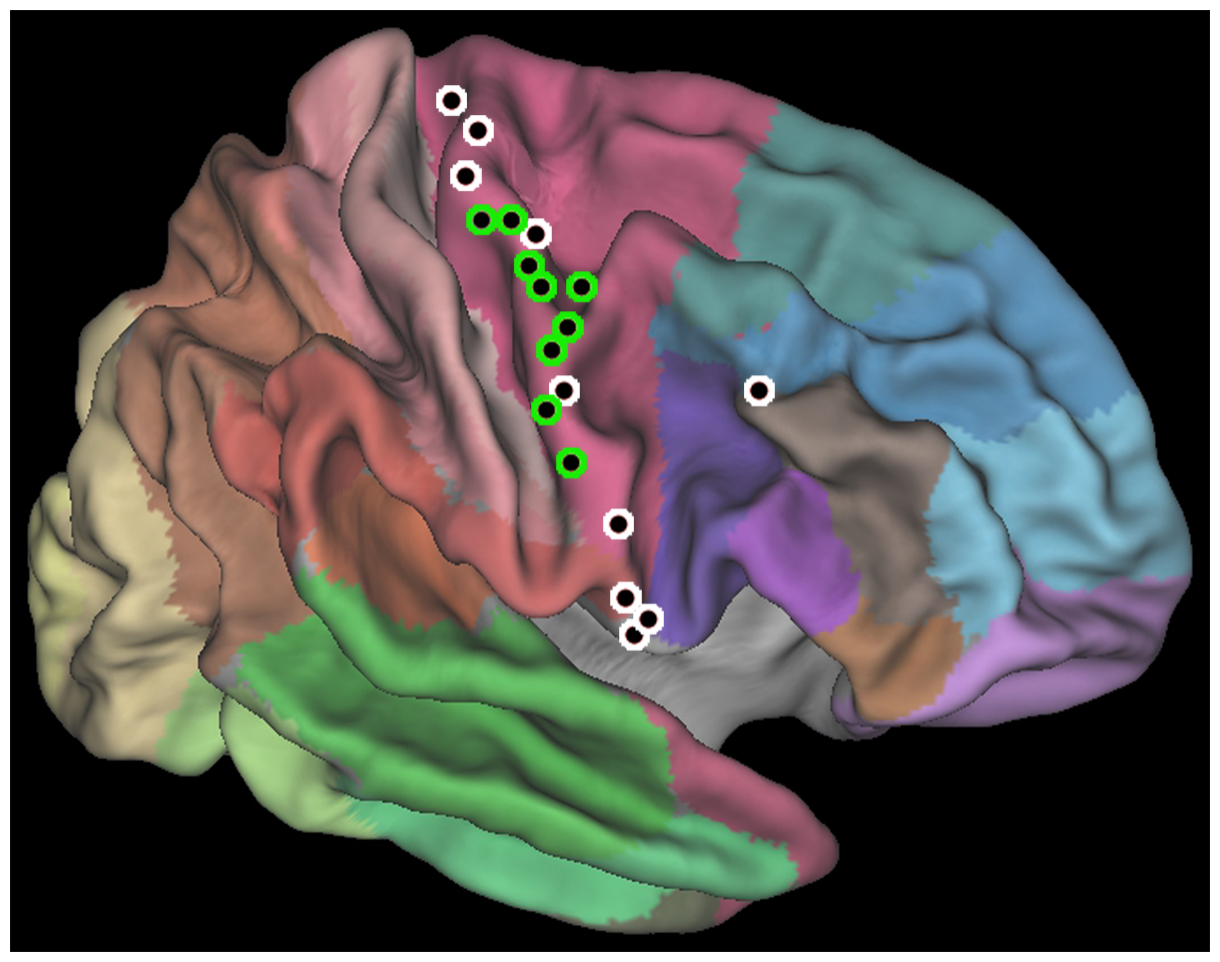
Illustration of the recording sites. Entrance point of the 19 electrodes implanted in the eight patients are plotted on an inflated PALS atlas surface according to their MNI coordinates. Brodmann areas are shown (Caret®; see [36]). Green: electrodes with at least one contact showing a significant effect of condition (BIO>PHY) in the gamma band. White: electrodes with no contacts showing a significant effect of condition. Sites are illustrated in the right hemisphere. All entrance point are localized in the precentral gyrus, with the only exception of a rostral electrode, approaching the deep PMC from the BA44. The localization of each of the 107 recording contact was assessed by the coregistration of pre-implantation volumetric brain MRI with post-implantation volumetric brain CT (Slicer®).

The analysis was performed on all the contacts located in the precentral region (n = 107), from 19 electrodes implanted in eight patients (see Figure 2 and Table 1 for the localization of the entrance points). For each contact located in the precentral region, we calculated the average power value relative to the baseline during the 4sec of stimulus presentation for all the experimental conditions: Biological Observation (OBS-BIO), Physical Observation (OBS-PHY), Biological Sequencing (SEQ-BIO) and Physical Sequencing (SEQ-PHY). All power values were subsequently log-transformed and expressed in dB. To assess the activity of PMC, we evaluated with a one-sample t-test the significance of gamma band power values relative to all conditions versus a zero-mean distribution. To reduce the false positive ratio, we considered as degree of freedom not the number of contact points (i.e. 107), but the number of patients (i.e. 8). A repeated measures ANOVA was performed per each contact, considering TASK (SEQ vs. OBS) and CONDITION (BIO vs. PHY) as factors. The TASK factor was aimed to assess the sequencing effect, and the CONDITION factor was aimed to assess the motor simulation effect. Beside the analysis on the single contacts, a population analysis for each of the three bands of interest (gamma = 50–150 Hz; alpha = 8–13 Hz; beta = 15–30 Hz) has been performed considering all the 107 recording sites. For each subsequent analysis, a repeated measures ANOVA was performed using CONDITION (BIO vs. PHY) and TASK (SEQ vs. OBS) as factors. Furthermore, to assess whether the gamma, alpha and beta band modulations were affected by the accuracy of the response, per each band we performed an additional repeated measures ANOVA using CONDITION (BIO vs. PHY) and ACCURACY (grouping together all the band modulation during the correct trials vs. band modulation during the wrong trials) as factors. Post-hoc analyses were conducted for each significant interaction by means of Bonferroni correction.

**Table 1:** Electrode entrance points.

Patient	Electrode	X	Y	Z	Hemisphere	Effect of Condition
P1	M'	–40.9	–8.3	61.1	Left	C
P1	N'	–59.3	–0.5	39.8	Left	C
P2	L'	–40.1	–17.8	65.6	Left	–
P2	R'	–57.8	–6.4	44.0	Left	C
P3	N	59.5	–5.4	41.4	Right	C
P3	R	64.5	8.6	12.1	Right	–
P4	H	58.7	4.2	38.1	Right	C
P4	M	44.1	–7.4	60.9	Right	C
P4	Z	18.1	–24.0	69.4	Right	–
P5	M	33.6	–13.1	65.1	Right	–
P5	N	52.8	5.7	43.2	Right	–
P6	N	58.1	–6.2	42.3	Right	–
P6	R	63.5	–2.5	25.8	Right	–
P7	M	45.0	–8.3	61.2	Right	C
P7	N	54.4	4.8	45.5	Right	C
P7	R	63.3	10.4	7.1	Right	–
P8	F	52.3	–6.8	51.7	Right	C
P8	M	35.4	–13.4	66.5	Right	–
P8	R	60.6	7.4	7.0	Right	–

The MNI coordinates of the entrance points and the side of implantation of each electrode are shown. The last column shows which electrode had at least one contact showing a significant effect of condition (BIO>PHY), as shown in Figure 2.

## Results

### Behavioral Data

During the sequencing block, behavioral results were collected from each patient to evaluate the error rate in the SEQ-BIO and SEQ-PHY conditions. Correct responses were given in 73% (SE ±3%) of all trials. In particular, SEQ-BIO was correctly sequenced in 64% (SE ±2) of cases, while SEQ-PHY was correctly sequenced in 79% (SE ±5%) of cases, showing a slightly higher error rate for the SEQ-BIO condition. A repeated measures ANOVA was conducted on the error rate, with RESPONSE (Correct vs. Wrong) and CONDITION (SEQ-BIO vs. SEQ-PHY) as main factors. Results showed that the effect of CONDITION was not statistically significant (F(1,7) = 1,0; p = 0.351).

### Induced Gamma band responses

**Figure 3:**
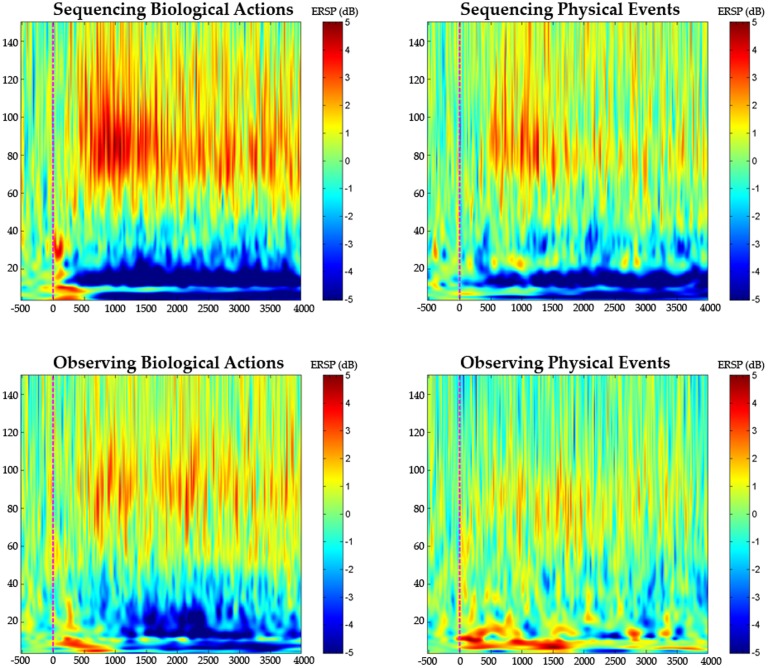
Time/Frequency maps. Results of the four investigated conditions from a representative contact showing a significant TASK*CONDITION interaction, with SEQ-BIO > SEQ-PHY in the gamma band (electrode F from P8). Time zero indicates the stimulus onset. All frequencies from 8 Hz to 150 Hz are shown. The segregation between alpha, beta and gamma frequency bands is clearly visible. Note the higher gamma power increase during the sequencing of the biological actions.

**Figure 4:**
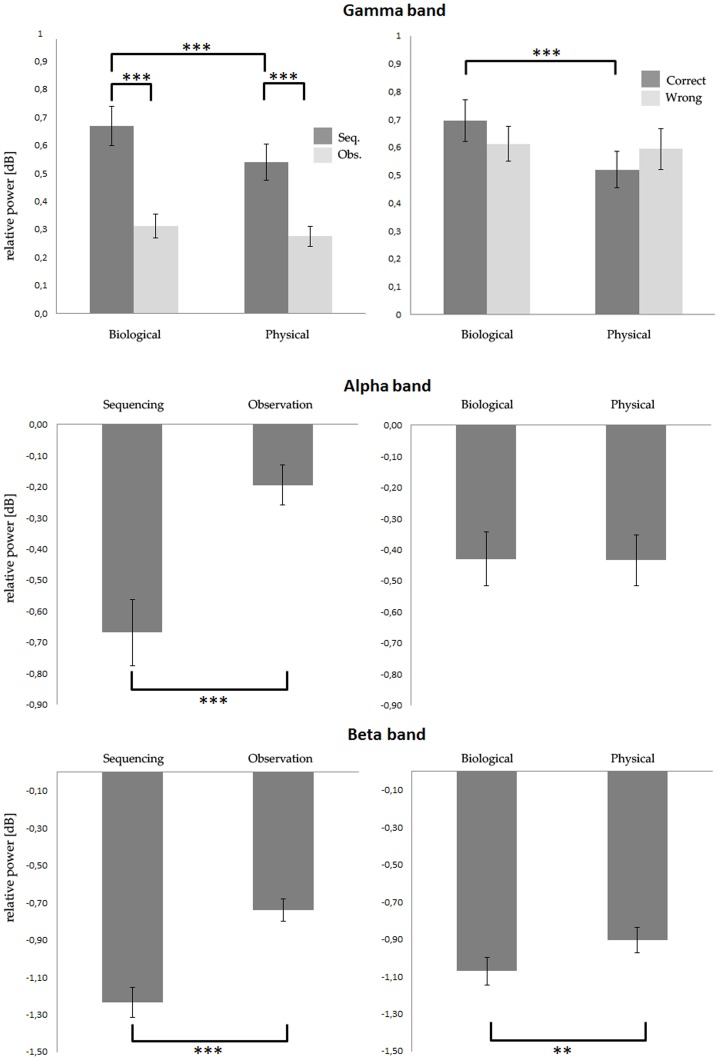
Statistical analysis conducted on the Gamma, Alpha and Beta bands. Gamma band. Left panel shows the results for the Condition (biological vs. physical) * Task (sequencing vs. observation) interaction. In particular, the sequencing of biological actions elicited a higher gamma modulation than the passive observation of the same stimuli (SEQ-BIO > OBS-BIO), as well as the sequencing of physical events (SEQ-BIO > SEQ-PHY). Main effects of Task and Condition are not shown. Right panel shows the effect of accuracy in the gamma modulation. No significant interaction was found between correct and wrong trials, in the biological condition, and a significant difference between biological and physical conditions in correct sequencing. Error bars indicate standard error. Horizontal bars indicate significant post-hoc interactions (*** p<0.0001). **Alpha and Beta bands**. The main effect of task (left) and conditions (right) are shown separately, given the lack of significant Condition*Task interaction. Note the lack of a main effect of condition, and the stronger effect of task, in the Alpha band. Error bars indicate standard error. Horizontal bars indicate significant results (** p<0.001; *** p<0.0001).

A first analysis was aimed to assess the responsiveness of PMC to the administered tasks. The one-sample t-test returned gamma power values significantly higher than zero for all four conditions (SEQ-BIO: p = 0,02; OBS-BIO: p = 0,04; SEQ-PHY: p = 0,03; OBS-PHY: p = 0,04), demonstrating that PMC is actively engaged in both tasks and conditions. Subsequently, we detected the task-related contacts among all the ones located in the precentral region (n = 107) in the eight patients. The repeated measures ANOVA showed that 100 out of 107 contacts (93.5%) had a significant main effect of TASK, and that 32 out of 107 contacts (29.9%) had a main effect of CONDITION. All these 32 contacts also exhibited a significant TASK effect. In contrast, 7 contacts (6.5%) did not show any significant main effect and were discarded from further analyses. As a third analysis, a repeated measures ANOVA with the same factors was performed grouping together all the 107 contacts. Results showed a significant effect of TASK (F(1,106) = 64,4; p<0.0001), indicating a stronger power increase during the sequencing block, as well as a significant effect of CONDITION (F(1,106) = 24,0; p<0.0001), indicating a stronger power increase during the biological condition. Furthermore, we found a statistically significant TASK*CONDITION interaction (F(1,106) = 13,5; p<0.0005; see Figure 3 and Figure 4). Post-hoc analysis showed a stronger gamma-band activity during the SEQ-BIO, as compared to SEQ-PHY, OBS-BIO and OBS-PHY (p<0.0001 for all comparisons). Furthermore, SEQ-PHY showed stronger gamma-band activity than OBS-PHY (p<0,0001) and OBS-BIO (p<0,0001). Biological observation was not statistically higher than physical observation (p = 0.21; see Figure 4).

### Independence of Gamma band responses from behavioral performance

To assess whether the gamma power increase during the SEQ-BIO was affected by the higher error rate, we performed a repeated measures ANOVA considering ACCURACY (Correct trials vs. Wrong trials) and CONDITION (BIO vs. PHY) as factors. Results showed a significant effect of CONDITION (F(1,106) = 21,8; p<0.0001), with a stronger power increase during the biological condition, and a significant RESPONSE*CONDITION interaction (F(1,106) = 11,6; p<0.001). In contrast, the main effect of ACCURACY was not significant (F(1,106) = 0,1; p<0.85). Post-hoc analysis showed that the increase of gamma power during the correct sequencing of biological actions was significantly higher than that during the correct sequencing of physical events (p<0.0001), but not different from that during the wrong sequencing of biological actions (p = 0.1; Figure 4).

### Alpha band desynchronization

The one-sample t-test performed on alpha band power values exhibited a trend for the sequencing task (SEQ-BIO: p = 0,07; SEQ-PHY: p = 0,07), while no significance for the observation one (OBS-BIO: p = 0,26; OBS-PHY: p = 0,24). The repeated measures ANOVA showed that 60 out of 107 contacts (56.1%) had a significant main effect of TASK, and that 38 out of 107 contacts (35.5%) had a main effect of CONDITION. In contrast, 26 contacts (24.3%) did not show any significant main effect and were discarded from further analysis. A repeated measures ANOVA with the same factors was performed grouping together all the 107 contacts. Results showed a significant effect of TASK (F(1,106) = 37,1; p<0.0001), indicating a stronger power suppression during the sequencing block. In contrast with the results obtained from the gamma band, the alpha band was similarly modulated by the biological and physical condition (F(1,106) = 0,005; p = 0.94; see Figure 4). The TASK*CONDITION interaction was not significant (F(1,106) = 0,1; p = 0.75). Furthermore, we found a significantly stronger power suppression during the correctly sequenced trials as compared to the wrong ones (F(1,106) = 59,9; p<0.0001), as well as a significant effect of CONDITION (F(1,106) = 11,9; p<0.0005).

### Beta band desynchronization

The preliminary assessment of beta band showed that significant p-values for all conditions (SEQ-BIO: p = 0,002; OBS-BIO: p = 0,007; SEQ-PHY: p = 0,004; OBS-PHY: p = 0,015). A repeated measures ANOVA showed that 76 out of 107 contacts (71.0%) had a significant main effect of TASK, and that 39 out of 107 contacts (36.4%) had a main effect of CONDITION. In contrast, 21 contacts (19.6%) did not show any significant main effect and were discarded from further analysis. A repeated measures ANOVA with the same factors was performed grouping together all the 107 contacts. Results showed a significant effect of TASK (F(1,106) = 73,2; p<0.0001), indicating a stronger power suppression during the sequencing block. Unlike the results from the alpha band, and similarly to those from the gamma band, the main effect of CONDITION was also significant (F(1,106) = 9,4; p<0.005), indicating a stronger power suppression during the sequencing of biological actions (Figure 4). The TASK*CONDITION interaction was not significant (F(1,106) = 0,1; p = 0.70). The ANOVA performed considering ACCURACY (Correct trials vs. Wrong trials) and CONDITION (BIO vs. PHY) showed a significant effect of ACCURACY (F(1,106) = 22,4; p<0.0001), similar to the results obtained from the alpha band.

## Discussion

In the present study we tested the reactivity of different brain rhythms to the sequencing of randomly presented images and to the passive observation of the same stimuli. The images were extracted from movies depicting biological actions and physical events, thus allowing us to dissociate the specific contributions of an event sequencing mechanism (meant as a sequencing > observation effect) to a motor simulation involved in the coding of biological stimuli (meant as a biological > physical effect). Imaging studies showed that both mechanisms activate the PMC but the coexistence of sequencing and off-line simulations within the same cortical region is still poorly understood. We found that these mechanisms are differentially represented in the three investigated frequency bands.

The gamma band showed a preference for the sequencing relative to the passive observation of the same stimuli, in line with evidence that PMC is involved in the sequencing and prediction of many kinds of dynamics [13] [14]. A possible explanation for this result and in particular for the significant gamma increase during event sequencing is that, as predicted by the HAPEM model [16], PMC houses styles of transformations, such as rotation, deformation, position translation, that could be detached from the motor output and exploited for both action and event sequencing. An alternative explanation is that many different variables are intrinsically part of the sequencing process and likely enhance the gamma activity. Following Tracy and collaborators [15] this process is solved by a comparator mechanism involved in the comparison the stimuli, holding the information in working memory and finally generating a tag that assigns the information a place in an ordered sequence. According to this alternative view, our data shows that this peculiar mix of attention, working memory, arousal and other variables involved by the correct sequencing of images, is reflected in the premotor gamma activity. More interesting, however, is the result that gamma frequency band also showed a preference for the coding of biological actions relative to physical events, in line with the view that biological actions are processed in the PMC in a variety of motor simulations, including motor planning, motor imagery or action observation [6] [7] [8]. It is noteworthy that the strongest response was elicited during the sequencing of biological actions, suggesting that PMC is maximally activated when action representations are triggered by visually presented stimuli and actively used to mentally simulate the action to be reconstructed, that is, when both event sequencing and motor simulation mechanisms are activated (see Figure 3). This result demonstrates that all the above mentioned variables involved in the sequencing but not in the passive observation, cannot explain the different modulation of the gamma responses in the sequencing task, given that decision-making, attention, working memory, arousal and motor preparation are constant among the biological and physical conditions. Viceversa, they strongly support a simulationist interpretation of our data.

It is unclear which specific simulation mechanisms are involved in PMC activation, and in particular whether the gamma activity observed was elicited by motor imagery [8] or the activation of the mirror system for action understanding [6], as has been suggested by other authors for similar action sequencing tasks [18]. Note that both action observation, typically triggering the mirror system, and mental reconstruction, requiring a motor imagery, are involved in completion of the action sequencing task. But the lack of a statistically significant preference for the passive observation of actions, as compared to that of physical events, suggests that the mirror system plays a minor role in action sequencing. Conversely, the synergistic effect of the event sequencing and motor simulation mechanisms, resulting in a SEQ-BIO > SEQ-PHY effect, supports the view that performance in our task depends on the employment of a motor imagery strategy, in which the subject mentally simulates the execution of the presented action to reorder the presented images. A similar synergistic effect of both sequencing and motor simulation mechanisms has also been recently found in the cerebellum. Cattaneo and coworkers [19] asked cerebellar patients to sequence biological and physical images, and found that patients perform worse than controls in both tasks, but the performance was much worse in the biological sequencing. The authors interpreted these data as the effect of a synergy between the role of the cerebellum in monitoring sequences of internally generated or external events and its role in the social and affective domain.

The analysis of slower oscillations in the alpha and beta band demonstrates that these diverse cognitive functions are supported by different neurophysiological mechanisms within the same region, according to previous evidence that the pattern in the gamma band is not fully reproduced in the alpha and beta bands [24]. In fact, while motor simulation effect (intended as BIO > PHY) is strongly reflected in the gamma band, many different variables affect the lower bands, suggesting that the gamma signal is functionally more specific than power modulations in other bands. The alpha band was mainly modulated by the sequencing task but did not show any preference for biological actions. We interpret this result as the effect of more aspecific factors involved in the sequencing task. As previously highlighted, the sequencing and the observation tasks differ in many aspects, including arousal, attention and working memory, that are all part of the sequencing process [15]. The view that all these diverse functions modulate the alpha band is supported by many EEG data demonstrating a role of this band in attentional processes [29] [30] [31] and in protecting working memory maintenance against distractors [32]. The view that the alpha band is modulated by a wider range of integrated elements, possibly including selective attention and working memory, is also supported by our finding that this band was particularly influenced by the accuracy, in contrast to the evidence that the gamma power elicited during correct and wrong trials was comparable. Furthermore, the involvement of attentional and working memory processes in the sequencing of both biological and non-biological events is supported by imaging data showing that besides the PMC, these tasks usually recruit a wide network of brain regions involved in these functions, such as the medial frontal regions (BA 8, 9; [15]).

The beta band showed a stronger power suppression during the sequencing task but, in contrast to the alpha band, was also modulated by the processing of biological actions, albeit the effect was milder than in the gamma band. The result that the motor simulation affects the beta, but not the alpha band, is in line with previous evidence from the mu-rhythm literature. MEG experiments on the suppression of the rolandic mu-rhythm during motor simulation, and in particular during action observation, showed that this rhythm consists of two main frequency components, roughly corresponding to the alpha and beta band [33] [34]. Hari [35] showed that the alpha and the beta components have two different cortical generators: the 20 Hz beta component is generated from the motor cortex, while the 10 Hz alpha component is parietal. Our data strongly support this view. In fact, as compared to the alpha band, the beta showed a higher functional correlation with the gamma band, which is considered to be the more reliable marker of local neuronal activity [20] [21]. In particular, beta and gamma showed a significant modulation for the biological stimuli that we did not find in the alpha. As a consequence, since scalp EEG technique has a limited access to the high frequency gamma band investigated in this work (50–150 Hz), our suggestion is that scalp EEG experiments investigating the covert stages of action representation should focus on modulation in the beta band which behaves as a negative counterpart of the precentral gamma activity.
